# The LOTUS domain is a conserved DEAD-box RNA helicase regulator essential for the recruitment of Vasa to the germ plasm and nuage

**DOI:** 10.1101/gad.297051.117

**Published:** 2017-05-01

**Authors:** Mandy Jeske, Christoph W. Müller, Anne Ephrussi

**Affiliations:** 1 Developmental Biology Unit, European Molecular Biology Laboratory, 69117 Heidelberg, Germany;; 2 Structural and Computational Biology Unit, European Molecular Biology Laboratory, 69117 Heidelberg, Germany

**Keywords:** DEAD-box RNA helicase Vasa, Oskar, TDRD5, TDRD7, germ plasm, nuage

## Abstract

DEAD-box RNA helicases play important roles in a wide range of metabolic processes. Regulatory proteins can stimulate or block the activity of DEAD-box helicases. Here, we show that LOTUS (Limkain, Oskar, and Tudor containing proteins 5 and 7) domains present in the germline proteins Oskar, TDRD5 (Tudor domain-containing 5), and TDRD7 bind and stimulate the germline-specific DEAD-box RNA helicase Vasa. Our crystal structure of the LOTUS domain of Oskar in complex with the C-terminal RecA-like domain of Vasa reveals that the LOTUS domain occupies a surface on a DEAD-box helicase not implicated previously in the regulation of the enzyme's activity. We show that, in vivo, the localization of *Drosophila* Vasa to the nuage and germ plasm depends on its interaction with LOTUS domain proteins. The binding and stimulation of Vasa DEAD-box helicases by LOTUS domains are widely conserved.

## Abstract (precis)

Jeske et al. show that LOTUS domains present in the germline proteins Oskar, TDRD5, and TDRD7 bind and stimulate the germline-specific DEAD-box RNA helicase Vasa. In vivo, the localization of *Drosophila* Vasa to the nuage and germ plasm depends on its interaction with LOTUS domain proteins.

RNA helicases are key enzymes involved in almost all RNA metabolic processes, such as RNA synthesis, processing, translation, and decay (Linder and Jankowsky 2011). DEAD-box proteins form the largest family of RNA helicases and are characterized by the strictly conserved sequence motif Asp–Glu–Ala–Asp (DEAD) (Linder et al. 1989). The core of DEAD-box RNA helicases is formed by two highly conserved RecA-like domains, often flanked by helicase-specific N-terminal and C-terminal extensions (Jankowsky and Fairman 2007). DEAD-box proteins can bind RNA in an ATP-driven manner and have been shown to separate short RNA duplexes and displace proteins from ssRNA (Jankowsky and Bowers 2006; Jankowsky and Putnam 2010). Within cells, most DEAD-box proteins do not function in isolation but are part of larger multicomponent assemblies in which they catalyze remodeling of higher-order ribonucleoprotein (RNP) complexes (Linder and Jankowsky 2011; Jarmoskaite and Russell 2014). Within such complexes, the activity of DEAD-box helicases can be stimulated or repressed by regulatory proteins (Ozgur et al. 2015).

The conserved DEAD-box RNA helicase Vasa plays diverse functions in germ cell formation and germline maintenance in animals (Raz 2000; Lasko 2013). During *Drosophila* oogenesis, Vasa localizes to two functionally distinct compartments in the egg chamber: the germ plasm in oocytes and the nuage in nurse cells (Mahowald 2001; Gao and Arkov 2013). Nurse cells are transcriptionally active and provide the growing oocyte with RNAs and proteins required for oocyte development and patterning of the future embryo (Johnstone and Lasko 2001). In the nuage, Vasa plays an essential role in the piRNA pathway (Malone et al. 2009; Xiol et al. 2014; Nishida et al. 2015), a retrotransposon defense mechanism that helps maintain genome integrity (Luteijn and Ketting 2013; Sato and Siomi 2013; Czech and Hannon 2016). The *Drosophila* germ plasm (or pole plasm) is assembled at the posterior tip of the oocyte and specifies the *Drosophila* germ cell precursors called pole cells, which form at the posterior pole during early embryogenesis (for review, see Mahowald 2001). In *Drosophila*, germ cell specification is coupled to abdominal patterning: Embryos that fail to assemble a functional pole plasm are devoid of pole cells, and the resulting larvae lack abdominal segments such that the mispatterned larvae arrest in early development and die. Assembly of the *Drosophila* pole plasm is induced by the protein Oskar (for review, see Lehmann 2016). Oskar is produced in two protein isoforms, of which the short form (Short Oskar) is essential for assembly of a functional pole plasm (Markussen et al. 1995). The Short Oskar isoform recruits Vasa, which also plays an essential role in the pole plasm (Hay et al. 1988; Breitwieser et al. 1996). The long isoform contains an N-terminal extension that—by a yet unknown mechanism—prevents Oskar from interacting with Vasa in vivo (Markussen et al. 1995; Breitwieser et al. 1996). Recently, we reported a physical interaction between Oskar and Vasa and showed that the interaction is mediated by Oskar's LOTUS (Limkain, Oskar, and Tudor containing protein**s** 5 and 7) domain (Jeske et al. 2015).

The LOTUS domain (also known as OST-HTH) is conserved in bacteria, fungi, animals, and plants and was originally suggested to bind to RNA (Anantharaman et al. 2010; Callebaut and Mornon 2010). In animals, its discovery in the germline proteins Oskar, Tudor domain containing 5 (TDRD5), TDRD7, and meiosis arrest female 1 (MARF1; also known as Limkain B) led to the domain name LOTUS (Anantharaman et al. 2010; Callebaut and Mornon 2010). Like Oskar, the proteins TDRD5, TDRD7, and MARF1 play critical roles in germ cell development in animals, but their molecular function is not known. In mice, MARF1 is oocyte-specific and required for meiotic progression, and MARF1 mutant mouse females are sterile (Su et al. 2012). In contrast, mammalian TDRD5 and TDRD7 have important roles during spermatogenesis, and TDRD5- or TDRD7-deficient males are sterile (Lachke et al. 2011; Tanaka et al. 2011; Yabuta et al. 2011). In *Drosophila*, the TDRD5 and TDRD7 orthologs Tejas and Tapas are jointly required for localization of Vasa to the nuage and play a role in piRNA-mediated retrotransposon silencing (Patil and Kai 2010; Patil et al. 2014). Moreover, Tejas and Tapas interact with Vasa in coimmunoprecipitation experiments, and, in the case of Tejas, the LOTUS domain is required for the interaction (Patil and Kai 2010; Patil et al. 2014). This observation, together with our finding that the Oskar LOTUS domain interacts with Vasa but not RNA (Jeske et al. 2015), raised the question of whether Vasa interaction might be a conserved function of LOTUS domains.

Here, we address this question directly and show that the LOTUS domains of Oskar, Tejas, and Tapas physically interact with Vasa. We further demonstrate that the LOTUS domain stimulates the Vasa DEAD-box helicase activity and that this function is conserved from insects to humans. We present the crystal structure of the Oskar LOTUS domain in complex with the C-terminal RecA-like domain of Vasa and show that the LOTUS domain occupies a novel binding surface on a DEAD-box helicase. Based on our finding that Vasa binding requires a particular C-terminal extension present in the LOTUS domains of Oskar, TDRD5, and TDRD7 but not in any of the LOTUS domains of MARF1, we divided the LOTUS domains into two subclasses; namely, extended LOTUS (eLOTUS) and minimal LOTUS (mLOTUS) domains. Finally, our mutational analysis in *Drosophila* revealed that Vasa recruitment to the nuage and pole plasm depends on its interaction with eLOTUS domains. Our analysis identified the eLOTUS domain as a novel DEAD-box RNA helicase regulator and sheds light on the function of LOTUS domain proteins in animals.

## Results

### The LOTUS–Vasa interaction is conserved

**Figure 1.**
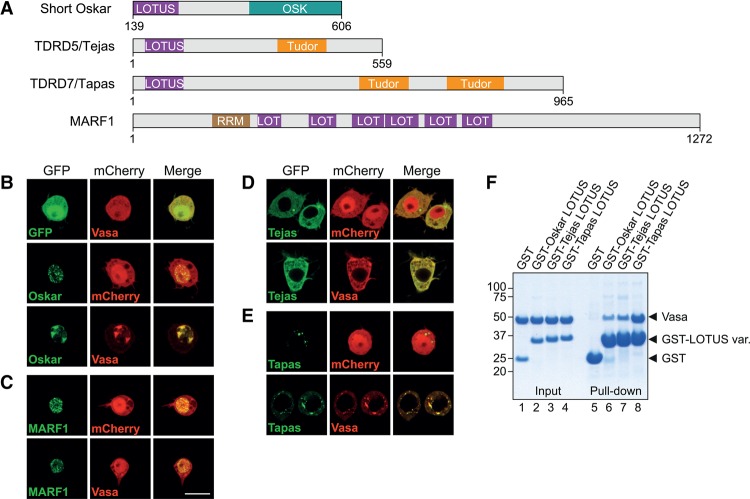
Vasa interacts with the LOTUS domains of Oskar, Tejas, and Tapas but not with MARF1. (*A*) Domain organization of *Drosophila* Oskar, TDRD5 (Tejas), TDRD7 (Tapas), and MARF1. In addition to LOTUS domains, Oskar contains an RNA-binding OSK domain, TDRD5 and TDRD7 contain one or more Tudor domains, and MARF1 contains one RNA recognition motif (RRM). The short isoform of Oskar is shown with residue numbers corresponding to those of the long isoform, which comprises the short isoform. (*B*–*E*) Plasmids encoding N-terminal GFP or mCherry fusions to the indicated proteins (in green or red) under the control of the actin 5C promoter were cotransfected into *Drosophila* S2R+ cells, grown for 2 d, and imaged by confocal microscopy. The full-length Short Oskar (*B*), MARF1 (*C*), Tejas (*D*), Tapas (*E*), and Vasa (*B*–*E*) were expressed. Bar, 10 µm. (*F*) GST pull-down assays using 10 µM GST or GST fusions of the LOTUS domains of Oskar, Tejas or Tapas and 20 µM Vasa 200–661. Inputs (lanes *1*–*4*) and immunoprecipitates (lanes *5*–*8*) were run on an SDS gel and stained with Coomassie brilliant blue. Protein markers (in kilodaltons) are indicated at the *left*. See also Supplemental Figure S1.

We reported previously a physical interaction between Oskar and Vasa and showed that Vasa interaction is mediated by the LOTUS domain of Oskar (Jeske et al. 2015). In animals, the LOTUS domain is also present in the germline proteins TDRD5, TDRD7, and MARF1 (Fig. 1A). To test whether these proteins are also able to bind Vasa, we used a colocalization assay in cultured *Drosophila* Schneider 2 R+ (S2R+) cells, which do not express Oskar and Vasa endogenously. When Short Oskar and Vasa were expressed as C-terminal fusions to either GFP or mCherry, transfected GFP-Oskar localized in speckles within the nucleus, while mCherry-Vasa was distributed ubiquitously in the cytoplasm and nucleus (Fig. 1B). Upon cotransfection, the localization of Oskar and Vasa changed drastically such that they colocalized within a few nuclear patches, indicating a direct Oskar–Vasa association (Fig. 1B). Similarly to GFP-Oskar, transfected GFP-MARF1 was also present in nuclear speckles in S2R+ cells (Fig. 1C). However, in contrast to Oskar, MARF1 did not influence the localization of Vasa, suggesting that these proteins do not interact. In contrast to Oskar and MARF1, GFP fusions to the *Drosophila* TDRD5 and TDRD7 orthologs Tejas and Tapas localized to the cytoplasm of S2R+ cells either uniformly (Tejas) or in speckles (Tapas). Upon cotransfection, Vasa was no longer distributed uniformly within the cytoplasm and nucleus but was recruited to sites of Tejas and Tapas localization (Fig. 1B,D,E), strongly suggesting that Vasa interacts with Tejas and Tapas. Interestingly, as in the case of Oskar, the Vasa interaction of Tejas and Tapas is mediated by their LOTUS domains, as constructs lacking the domain did not drive Vasa relocalization (Supplemental Fig. S1). The direct interaction of the LOTUS domains of Tejas and Tapas with Vasa was also observed in GST pull-down assays performed with purified proteins (Fig. 1F), with the Tapas LOTUS domain showing the highest affinity for Vasa. Taken together, our experiments demonstrate that Vasa interacts with not only Oskar but also Tejas and Tapas and, in each case, via the LOTUS domain of the protein.

### The LOTUS domain of Oskar interacts with Vasa's C-terminal RecA-like domain

**Figure 2.**
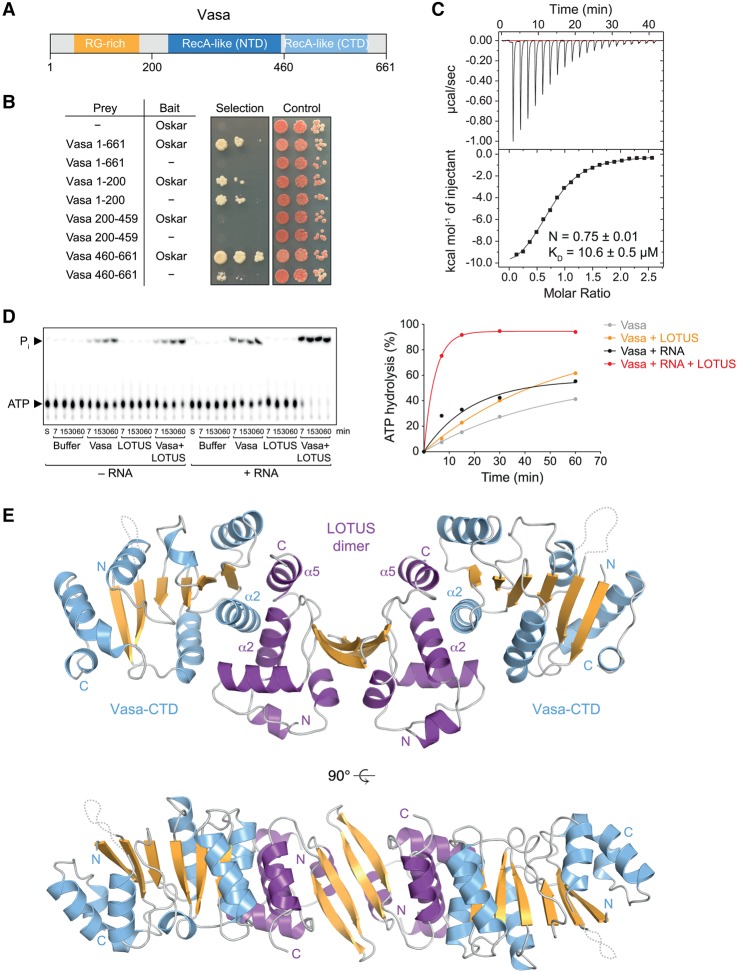
Crystal structure of the Oskar LOTUS–Vasa-CTD complex. (*A*) Vasa protein domain organization. (*B*) Yeast two-hybrid assays using prey constructs containing the indicated Vasa fragments or no insertion (−). The bait constructs contained full-length Short Oskar or no insertion (−). Three 10-fold dilutions of the cells were spotted. The selection medium lacked histidine, and positive growth on the selection medium indicates interaction. (*C*) ITC data of titration of Vasa 463–661 to the Oskar LOTUS domain (amino acids 144–240). Please note that the LOTUS domain of Oskar forms dimers (Jeske et al. 2015). The curve was fitted using the LOTUS monomer concentration. (*D*) ATPase time courses using 20 µM Vasa 200–661 in the absence or presence of 50 µM RNA oligo and/or 400 µM Osk 144–240. Original thin-layer chromatography (TLC; *left* panel) and quantification (*right* panel) are shown. The image is the result of one experiment and represents an assembly of several TLC plates that were exposed simultaneously to one phosphorimager screen. These and all subsequent ATP hydrolysis data were fit to an exponential solely to guide the eye of the reader. (*E*) Crystal structure of the complex consisting of the LOTUS domain dimer of Oskar (amino acids 139–240) and two Vasa C-terminal RecA-like domains (amino acids 463–623). The twofold symmetry of the model is noncrystallographic. See also Supplemental Table S1.

Vasa comprises a helicase core composed of an N-terminal and a C-terminal RecA-like domain, which is preceded by an N-terminal arginine/glycine (RG)-rich extension that is predicted to be disordered (Fig. 2A). To identify the region of Vasa that interacts with LOTUS domains, we performed yeast two-hybrid assays on Short Oskar and three individual Vasa domains corresponding to amino acids 1–200 (RG-rich region), amino acids 200–460 (Vasa-NTD [N-terminal domain]), and amino acids 461–661 (Vasa-CTD [C-terminal domain]). We thus found that Oskar interacts with full-length Vasa as well as with the Vasa-CTD but not the RG-rich region or Vasa-NTD (Fig. 2B). Isothermal titration calorimetry (ITC) experiments using purified proteins revealed a dissociation constant (*K*_D_) of ∼10 µM for the LOTUS–Vasa-CTD complex (Fig. 2C). We previously determined a similar *K*_D_ for a complex consisting of the LOTUS domain and the full Vasa helicase core (Jeske et al. 2015). This suggests that the complex consisting of the LOTUS domain and the Vasa-CTD forms the minimal unit of the Oskar–Vasa interaction.

Oskar binding to Vasa was mapped previously not to the Vasa-CTD but to regions for which we detected no interaction with Oskar. In one study, Oskar was shown to bind to a stretch residing in the Vasa-NTD (amino acids 200–252) (Anne 2010). However, in these experiments, the Vasa-CTD alone was not tested, and the Vasa-NTD was cut into pieces, most likely leading to unfolding and exposure of hydrophobic patches of the fragments, possibly resulting in stickiness. A second study reported Oskar binding to a region of Vasa C-terminal to the helicase core; however, the basis of this conclusion is unclear (Kirino et al. 2010). As the presence or absence of this C-terminal Vasa extension did not result in a difference in LOTUS binding to Vasa in our experiments (Jeske et al. 2015; see also the crystal structure below), we conclude that the region outside the helicase core is probably irrelevant for LOTUS binding.

### The LOTUS domain of Oskar stimulates Vasa helicase activity

We asked whether the binding of the LOTUS domain of Oskar to Vasa might modulate Vasa's DEAD-box helicase activity. Studies of several different DEAD-box proteins have defined the individual enzymatic steps of the helicase cycle (for review, see Jankowsky 2011). In an unbound state, the N-terminal and C-terminal RecA-like domains of DEAD-box helicases can move freely toward each other. ATP binding increases the affinity of the helicase for RNA, and, upon RNA binding, the helicase adopts a closed conformation. In the closed form, the bound RNA is bent, leading to local strand separation of an RNA duplex and release of one strand from the helicase. Last, upon ATP hydrolysis, the second RNA strand is released. As ATP hydrolysis is a late step in the DEAD-box helicase cycle, we used ATPase assays as an indirect measure of the helicase activity of Vasa.

To so do, we incubated trace amounts of [γ-^32^P] ATP with the Vasa helicase core and separated the hydrolysis products ADP and γ-^32^P_i_ from the substrate by thin-layer chromatography (TLC). For simplicity, the experiments shown contained only ssRNA, as we did not observe a difference in ATPase activity whether ssRNA or dsRNA was present. Incubating ATP with high Vasa concentrations (20 µM) revealed a low level of ATP hydrolysis activity (Fig. 2D, gray curve). The ATP hydrolysis activity of Vasa was enhanced in the presence of an RNA oligonucleotide (30 µM) (Fig. 2D, black curve), consistent with the previous demonstration of a stimulating effect of RNA on the ATPase activity of DEAD-box proteins (Jankowsky 2011). When the LOTUS domain of Oskar (400 µM) was incubated with Vasa in the absence of RNA, a slight increase in Vasa activity was also observed (Fig. 2D, cf. gray and orange curves). However, when the LOTUS domain was incubated with Vasa in the presence of RNA, the stimulation of the ATPase activity was very strong (Fig. 2D, red curve). Control experiments show that the LOTUS protein preparation was free of contaminating ATPases (Fig. 2D). This experiment shows that the LOTUS domain of Oskar not only mediates an interaction with Vasa but also acts as a stimulator of Vasa's enzymatic activity.

### Crystal structure of the LOTUS–Vasa complex

The individual crystal structures of the Vasa helicase core and Oskar LOTUS domain were determined previously (Sengoku et al. 2006; Jeske et al. 2015; Yang et al. 2015). Furthermore, we reported that the LOTUS domain of the Oskar protein of a few insects forms dimers but that dimerization is not a uniformly conserved feature of LOTUS domains (Jeske et al. 2015). To obtain structural information regarding the LOTUS–Vasa complex and the role of LOTUS in stimulating Vasa's ATPase activity, we carried out cocrystallization experiments using the LOTUS domain of Oskar (amino acids 139–240) and the Vasa-CTD lacking the C-terminal extension (amino acids 463–623). The crystals that we obtained diffracted to 1.4 Å resolution, and the structure was solved by molecular replacement and subsequently refined against the data set to *R*/*R*_free_ values of 16.9%/19.9% (see Supplemental Table S1 for data collection and model refinement statistics). The crystal structure consists of one LOTUS dimer and two molecules of Vasa-CTD, each one bound to a LOTUS monomer on the side opposite to the dimerization interface (Fig. 2E). Hence, Vasa binding does not affect dimerization of the LOTUS domain, which is consistent with the experimentally determined 1:1 stoichiometry of the LOTUS–Vasa complex (Jeske et al. 2015). The interface area of one LOTUS monomer–Vasa-CTD subcomplex measures 1080 Å^2^ (PISA analysis) (Krissinel and Henrick 2007).

**Figure 3.**
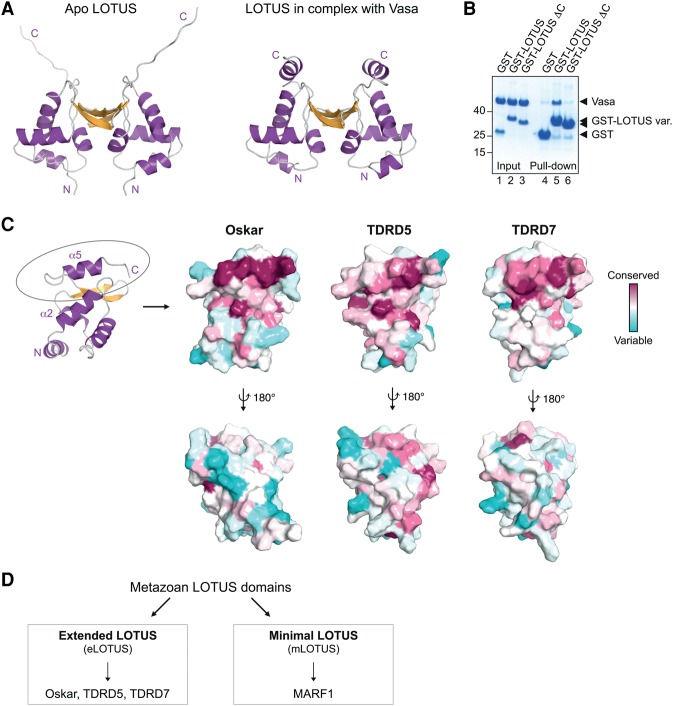
Vasa interaction requires a LOTUS domain C-terminal extension. (*A*) Modified model of the previously solved Oskar LOTUS domain dimer (*left* panel) in comparison with the dimer found in complex with Vasa (*right* panel). In the original apo LOTUS dimer structure (Protein Data Bank [PDB] 5A48) (Jeske et al. 2015), specific crystal contacts allowed the detection of electron density for the unstructured C-terminal extension in one of the two subunits. The apo LOTUS dimer shown here was created using two copies of this extended subunit. (*B*) GST pull-down assays using 10 µM GST or GST-Oskar LOTUS containing (amino acids 139–240) or lacking (ΔC; amino acids 139–222) the C-terminal extension and 20 µM His-Vasa 200–661. Input and eluates were run on an SDS gel and stained with Coomassie brilliant blue. Protein markers (in kilodaltons) are indicated at the *left*. (*C*) Surface representation of the LOTUS domain of Oskar (monomer; *left*), TDRD5 (*middle*), or TDRD7 (*right*) colored according to residue conservation (Ashkenazy et al. 2016). (*Top* row) The C-terminally extended α helix is highlighted by an ellipse in the cartoon representation and is the most conserved part of the LOTUS domains. (*Bottom* row) Conservation of the dimer interface of the Oskar LOTUS domain of Oskar is not obvious in this surface analysis, as Oskar dimerization occurs only in drosophilids and a few other insects. For the analysis of the LOTUS domains of TDRD5 and TDRD7, models of the Tejas or Tapas LOTUS domains were generated using SWISS-MODEL (Biasini et al. 2014) and the Oskar LOTUS domain monomer as template. (*D*) LOTUS domains can be divided into two subclasses depending on the presence (eLOTUS) or absence (mLOTUS) of the C-terminal extension.

In the complex, the LOTUS domain is contacted mainly via the α2 helix of the Vasa-CTD (Fig. 2D). Conversely, the Vasa-CTD is contacted by two helices of the LOTUS domain: the α2 helix, which is part of the trihelical bundle of the winged HTH (wHTH) core, and the α5 helix, which is positioned at the LOTUS domain C terminus (Fig. 2E). Interestingly, although one of our previously obtained crystal structures of the LOTUS domain alone also contained the C-terminal extension (α5 helix in the cocrystal), the extension did not adopt a particular secondary structure but most likely was disordered (Fig. 3A; Jeske et al. 2015). This suggests that the LOTUS domain C-terminal extension forms an α helix upon interaction with Vasa.

### Two distinct subclasses of LOTUS domains

Vasa interacts with the LOTUS domains of Oskar, Tejas, and Tapas but not the LOTUS domain protein MARF1 (Fig. 1). The difference in Vasa binding can be explained by a structural difference between the LOTUS domains. While the LOTUS domains of Oskar, TDRD5, and TDRD7 all comprise a C-terminal extension, the LOTUS domains of MARF1 do not (Callebaut and Mornon 2010). We tested the importance of the C-terminal LOTUS extension for Vasa binding in a GST pull-down assay. This revealed that a LOTUS domain lacking the extension (“LOTUSΔC”) was not able to interact with Vasa (Fig. 3B), showing that presence of the C-terminal extension is essential for Vasa interaction. However, the LOTUS C-terminal extension alone does not interact with Vasa (M Jeske, unpubl.), indicating that both the α2 and α5 helices of the LOTUS domain are required for interaction with Vasa. Nevertheless, analysis of the Oskar LOTUS domain structure and of structural models of the TDRD5 and TDRD7 LOTUS domains reveals the highest surface conservation at the α5 helix (Fig. 3C), highlighting its functional importance and suggesting that Vasa interaction is a conserved function of the LOTUS domains of Oskar, TDRD5, and TDRD7. Furthermore, these analyses suggest that the LOTUS domains of Oskar, TDRD5, and TDRD7 bind to Vasa with an equivalent surface. Based on the structural and functional differences between LOTUS domains that either contain or lack the C-terminal extension, we propose the division of this domain family into eLOTUS domains present in Oskar, TDRD5, and TDRD7 and mLOTUS domains present in MARF1 (Fig. 3D).

### LOTUS–Vasa interface mutations

**Figure 4.**
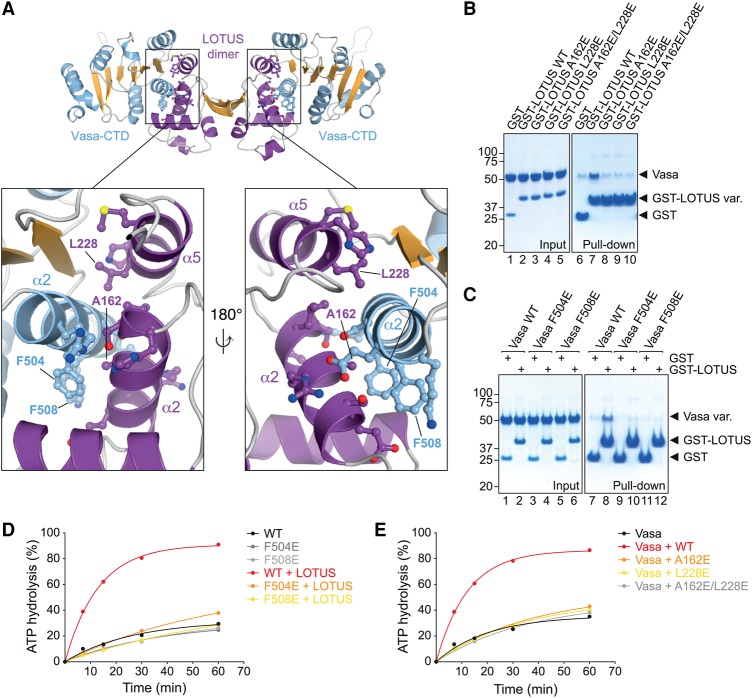
The LOTUS–Vasa interface. (*A*) Close-up view of the eLOTUS–Vasa interface. Residues that establish side chain-specific contacts within the interface are highlighted in a ball and stick representation. The residues that were mutated in subsequent experiments are labeled. (*B*) GST pull-down assays using 8.75 µM GST, wild-type or mutant Oskar GST-LOTUS as indicated, and 20 µM His-tagged Vasa 200–661. Samples from the experiment were run on an SDS gel and stained with Coomassie brilliant blue. Protein markers (in kilodaltons) are indicated at the *left*. (*C*) Experiment as in *B* using 8.75 µM GST or Oskar GST-LOTUS and 17.5 µM wild-type or mutant His-tagged Vasa 200–661 as indicated. (*D*,*E*) ATPase time courses in the presence of 10 µM RNA oligo and 5 µM wild-type or mutant His-Vasa 200–661 as indicated with or without 20 µM Oskar 144–240 (LOTUS) (*D*) or 5 µM His-Vasa 200–661 and 20 µM wild-type or mutant His-Oskar 139–240 as indicated (*E*). See Supplemental Figure S5 for the original TLC plates that were quantified to create the plots. See also Supplemental Figures S2–S4.

In order to validate the Vasa–eLOTUS interface and design mutant transgenes for in vivo analysis of the Vasa interaction with LOTUS domain proteins (see below), we aimed to identify point mutations that lie in the interface of the complex (Fig. 4A). All of the mutations tested in the following do not interfere with folding or the thermal stability of the proteins (Supplemental Fig. S2).

In Oskar, we identified one mutation each in the α2 helix (A162E) and α5 helix (L288E) of the eLOTUS domain that prevents its interaction with Vasa in GST pull-down assays (Fig. 4B). The residues A162 and L228 are not strictly conserved in eLOTUS domains (Supplemental Fig. S3A). The residue 162 bears a small side chain, which is either an alanine in the eLOTUS domains of Oskar and various TDRD7 proteins or a serine in TDRD5 proteins. The residue 228 is small and hydrophobic and either a leucine in the eLOTUS domain of Oskar or an isoleucine in the eLOTUS domains of TDRD5 and TDRD7 proteins. Together, this suggests that the eLOTUS–Vasa interaction tolerates little variation of the LOTUS interface residues.

In Vasa, we identified two point mutations in the α2 helix of the CTD (F504E and F508E), each of which prevents the Vasa–eLOTUS interaction in GST pull-down assays (Fig. 4C). F504 and F508 are highly conserved among Vasa proteins. The phenylalanine at position 504 is invariant, and the residue 508 is strictly aromatic (phenylalanine or tyrosine) in proteins from insects to humans (Supplemental Fig. S3B). Belle, the DEAD-box protein with the highest sequence similarity to Vasa, also contains an aromatic residue at the position equivalent to residue 508 in Vasa (Supplemental Fig. S4). However, Belle does not interact with Oskar, suggesting that a single aromatic residue is not sufficient for LOTUS domain binding, which is consistent with our mutational analysis of Vasa (Supplemental Fig. S4). We conclude that LOTUS domains bind specifically to the Vasa helicase.

We next tested the Oskar–Vasa interface mutations in Vasa ATPase assays. These and all subsequent reactions were performed in the presence of RNA. Vasa helicase cores that contain the F504E or F508E point mutation showed ATPase activity similar to that of wild-type proteins (Supplemental Fig. S5A), strongly suggesting that the mutations do not interfere with the proper folding of Vasa. However, the activity of the Vasa mutant proteins was not stimulated by the eLOTUS domain of Oskar (Fig. 4D). Similarly, the eLOTUS A162E and L228E single- and double-point mutant variants did not stimulate the ATPase activity of wild-type Vasa efficiently (Fig. 4E). Together, these experiments demonstrate that efficient stimulation of Vasa requires interaction with the eLOTUS domain via the interface that we identified.

### Vasa localization to germ plasm and the nuage depends on LOTUS domain interactions

**Figure 5.**
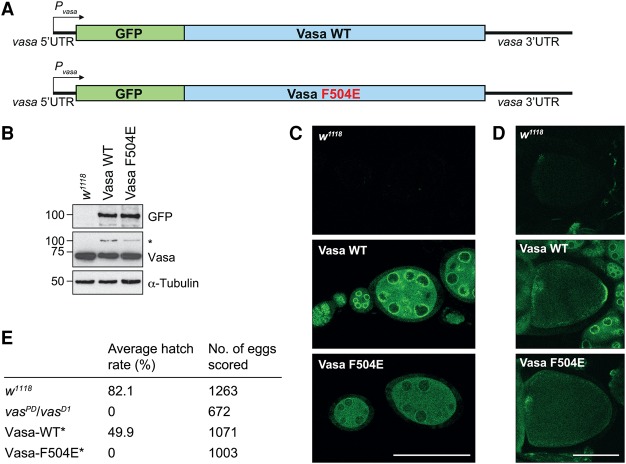
Vasa localization to germ plasm depends on LOTUS domain interactions. (*A*) Scheme of the Vasa wild-type and F504E mutant transgenes. “P” indicates the promoter. (*B*) Western blot analysis of transgene expression levels in *Drosophila* ovaries using antibodies against the proteins indicated. The transgenes were expressed in a wild-type background; hence, the anti-Vasa antibody recognizes endogenous Vasa and transgenic Vasa (*). (*C*,*D*) Transgenic Vasa-GFP was imaged by confocal microscopy. Young egg chambers (stages 1–7) (*C*) and oocytes (stage 10) (*D*) are shown. The egg chambers were imaged with identical microscope settings, and wild-type egg chambers (*w*^*1118*^) served as background controls. Bar, 100 µm. (*E*) Hatching rates of eggs laid by mothers of the indicated genotypes. (*) The transgenes were expressed in the *vasa^PD^*/*vasa^D1^* background.

In the *Drosophila* egg chamber, Vasa localizes to the nuage in the nurse cells and to the pole plasm in the oocyte. Pole plasm localization of Vasa depends on Short Oskar (Breitwieser et al. 1996), and localization of Vasa to the nuage depends on TDRD5 (Tejas) and TDRD7 (Tapas) (Patil et al. 2014). To study whether direct interaction between Vasa and the various LOTUS domain proteins is required for Vasa recruitment to the nuage and pole plasm, we analyzed in *Drosophila* the F504E mutation in Vasa that prevents the protein from binding to the Oskar LOTUS domain in vitro (Fig. 4). Importantly, this mutation does not affect Vasa's ATPase activity (Supplemental Fig. S5A). We generated transgenes that encode GFP fused to either wild-type or mutant Vasa, resulting in GFP-Vasa-WT and GFP-Vasa-F504E proteins, respectively (Fig. 5A). The transgenes were placed under the control of *vasa* regulatory sequences. Western blot analysis of ovary samples revealed that the different transgenes express GFP-Vasa-WT and GFP-Vasa-F504E to similar levels (Fig. 5B). However, compared with endogenous Vasa, the levels of expression of the transgenic protein were reduced (Fig. 5B), as reported previously for other *vasa* transgenes (e.g., Johnstone and Lasko 2004; Xiol et al. 2014; Dehghani and Lasko 2015).

Analysis of the localization of the GFP-Vasa fusion proteins in *Drosophila* egg chambers revealed that, similar to endogenous Vasa, GFP-Vasa-WT concentrates in the nuage, surrounding the nurse cell nuclei, and at the posterior pole of oocytes (Fig. 5C,D). In contrast, in the nurse cells, the enrichment of GFP-Vasa-F504E to the nuage was strongly reduced, and, in oocytes, GFP-Vasa-F504E did not localize to the pole plasm but was ubiquitously distributed (Fig. 5C,D). This suggests that recruitment of Vasa to pole plasm and the nuage depends on its direct interaction with the LOTUS domains of Oskar and of TDRD5 and TDRD7, respectively.

Next, we assessed the ability of the Vasa transgenes to rescue the mutant *vasa*^*PD*^/*vasa*^*D1*^ phenotype using a hatching assay. Female flies that carry the *vasa*^*PD*^/*vasa*^*D1*^ alleles produce embryos that lack abdominal segments and fail to hatch (Fig. 5E; Lasko and Ashburner 1988). While the GFP-Vasa-WT transgene rescued the mutant phenotype to a great extent (∼50% hatching), the GFP-Vasa-F504E transgene did not (0% hatching) (Fig. 5E). This indicates that the sole presence of an active Vasa helicase in the egg chamber does not suffice for proper embryonic development, and interaction with a LOTUS domain protein is required.

### Vasa stimulation by eLOTUS domains is conserved across different species

**Figure 6.**
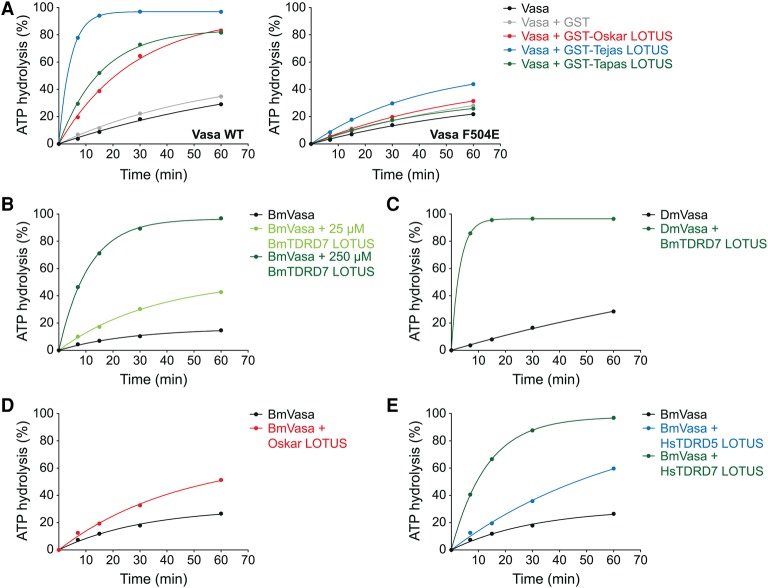
The LOTUS domain is a conserved Vasa stimulator. ATPase time courses using 5 µM indicated Vasa construct, 10 µM RNA oligo, and 20 µM indicated eLOTUS domain construct unless specified otherwise: wild-type (*left* panel) or F504E mutant (*right* panel) His-Vasa 200–661 and GST or GST fusions of the eLOTUS domain of Oskar, Tejas, or Tapas (*A*), 3 µM *Bombyx* Vasa 135–564 ± two different concentrations of *Bombyx* His-TDRD7 eLOTUS as indicated (*B*), *Drosophila* His-Vasa 200–661 ± *Bombyx* His-TDRD7 eLOTUS (*C*), *Bombyx* Vasa 135–564 ± *Drosophila* His-Oskar eLOTUS (amino acids 139–240) (*D*), and 5 µM *Bombyx* Vasa 135–564 ± 150 µM human His-TDRD5 eLOTUS or human His-TDRD7 eLOTUS (*E*). See Supplemental Figure S5 for the original TLC plates that were quantified to create the plots.

Vasa interacts with the eLOTUS domain of Oskar as well as the eLOTUS domains of Tejas and Tapas (Fig. 1), and its helicase activity is stimulated by the eLOTUS domain of Oskar (Fig. 2D). Interestingly, when we also tested the eLOTUS domains of Tejas and Tapas in the Vasa activity assays, we found that they also stimulate Vasa ATPase activity (Fig. 6A, left plot). Notably, the highest stimulation was observed with the eLOTUS domain of Tejas, although it did not show the highest affinity to Vasa in binding assays (Fig. 1F). The eLOTUS domains of Tejas and Tapas were unable to stimulate the activity of F504E mutant Vasa (Fig. 6A, right plot), which, together with the in vivo data (Fig. 5), suggests that the eLOTUS domains of Tejas, Tapas, and Oskar bind to the same surface on Vasa.

We also tested the Vasa and eLOTUS orthologs of the silk moth *Bombyx* in activity assays. Like the *Drosophila* proteins, the eLOTUS domain of *Bombyx* TDRD7 stimulated the activity of the *Bombyx* Vasa helicase core (Fig. 6B). Moreover, *Bombyx* Vasa was stimulated by the *Drosophila* Oskar eLOTUS domain, and, conversely, *Drosophila* Vasa was stimulated by the *Bombyx* TDRD7 eLOTUS domain (Fig. 6C,D). Finally, we wished to test the human orthologs of eLOTUS and Vasa in the ATPase assays. Unfortunately, the human Vasa helicase core was not soluble upon recombinant expression (M Jeske, unpubl.). We therefore tested the effect of the eLOTUS domains of human TDRD5 and TDRD7 on the activity of insect Vasa helicase cores. While the human eLOTUS domains stimulated *Bombyx* Vasa (Fig. 6E), they did not increase the ATPase activity of the *Drosophila* ortholog (M Jeske, unpubl.).

Taken together, our functional assays demonstrate that the eLOTUS domain is a widely conserved positive regulator of Vasa. This is remarkable considering the low sequence identity among eLOTUS domains (Supplemental Fig. S3A).

### eLOTUS is a novel DEAD-box helicase regulator

**Figure 7.**
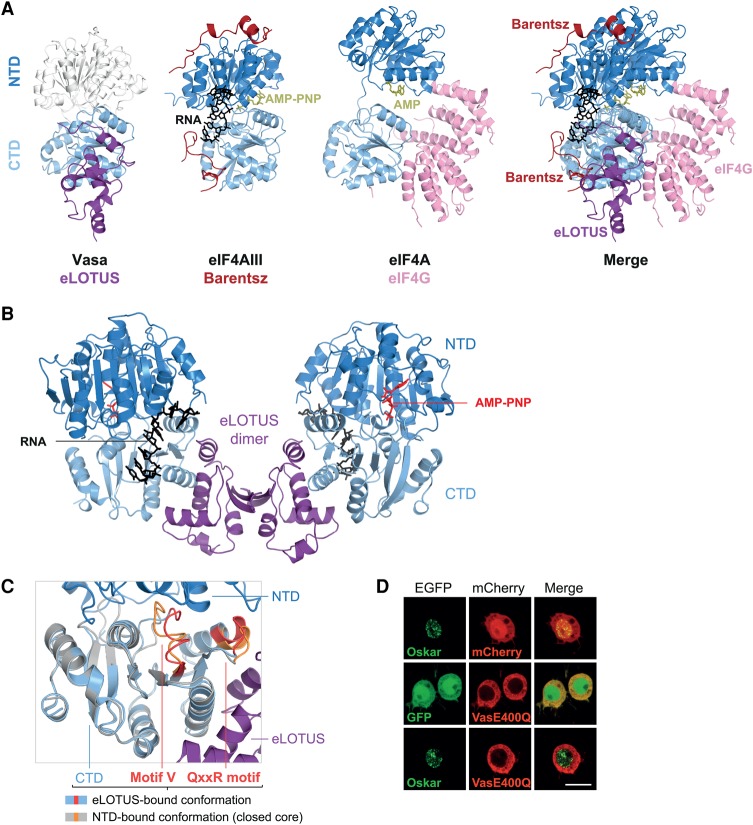
LOTUS is a novel DEAD-box RNA helicase regulator. (*A*) Comparison of the eLOTUS–Vasa complex with the Barentsz–eIF4AIII (PDB 2HYI) and eIF4G–eIF4A (PDB 2VSO) complexes. eIF4G is a MIF4G domain protein. All complexes are oriented with respect to their CTDs (light blue). The NTD of Vasa (white) was modeled onto the eLOTUS–CTD complex with the help of the helicase core structure (PDB 2DB3). Bound substrates are indicated. (*B*) Model of the eLOTUS dimer of Oskar in complex with two Vasa helicase cores bound to AMP-PNP and an RNA oligo (closed conformation). The Vasa core (PDB 2DB3) was superimposed based on the CTDs. (*C*) Detailed view of the superimposition of the CTD bound to eLOTUS and the CTD bound to the NTD (PDB 2DB3). The eLOTUS–CTD complex is colored purple (LOTUS) and light blue (CTD), and the NTD–CTD complex (closed helicase core) is colored in dark blue (NTD) and gray (CTD). The motifs QxxR and V are colored red in the eLOTUS–CTD complex and orange in the closed core. (*D*) Colocalization analysis in S2R+ cells. In contrast to GFP-Short Oskar and mCherry-Vasa, which colocalize in in S2R+ cells (see Fig. 1B), GFP-Short Oskar does not colocalize with mCherry-Vasa E400Q. The same result was obtained using a mutant Oskar protein variant that localizes in the cytoplasm (Supplemental Fig. S6). The experiments were performed in parallel with the one shown in Figure 1B. Bar, 10 µm.

To get further insight into how the eLOTUS domain of Oskar stimulates the helicase activity of Vasa, we compared the structure of the eLOTUS–Vasa-CTD complex with previously determined structures of DEAD-box helicases in complex with activating proteins (Fig. 7A; for review, see Ozgur et al. 2015). For example, the RNA-binding protein Barentsz/MLN51 contacts both RecA-like domains of the DEAD-box helicase eIF4AIII and stimulates helicase activity by contributing to RNA binding (Ballut et al. 2005; Andersen et al. 2006; Bono et al. 2006; Noble and Song 2007). In contrast to Barentsz, the eLOTUS domain of Oskar displayed no detectable RNA-binding activity (Jeske et al. 2015) and did not increase the affinity of Vasa for RNA (M Jeske, unpubl.). Another class of DEAD-box helicase stimulator that does not bind to RNA is the MIF4G domain. MIF4G domains stimulate the ATPase activity of their respective helicases by contacting both RecA-like domains and orienting them favorably for catalysis (Schütz et al. 2008; Montpetit et al. 2011; Chen et al. 2014; Mathys et al. 2014). In contrast to MIF4G domains and Barentsz, the eLOTUS domain contacts only the C-terminal RecA-like domain of the helicase. Moreover, the position of the eLOTUS domain on the CTD does not overlap with the positions of Barentsz or MIF4G binding to helicases (Fig. 7A, merge). Together, this suggests that the eLOTUS domain modulates Vasa helicase activity via a mechanism that differs from those of other known stimulators.

Superimposition of Vasa-CTD in the structure of our eLOTUS–CTD complex with the Vasa-CTD in the closed Vasa helicase core bound to RNA and a nonhydrolyzable ATP analog (Sengoku et al. 2006) resulted in a model of the eLOTUS dimer bound to two closed Vasa cores (Fig. 7B). This revealed that binding of the eLOTUS domain does not interfere sterically with the accessibility of ATP or RNA to the helicase. In this superimposition, the two CTD structures aligned well, with the exception of the DEAD-box helicase motifs QxxR and V (Fig. 7C). The motifs QxxR and V are involved mainly in interactions with the NTD, and the positions of both motifs are different in open and closed DEAD-box helicase structures (Sengoku et al. 2006). In the eLOTUS-bound CTD, the conformation of these motifs resembles that observed in DEAD-box helicases in their open state, suggesting that the conformation of the open state CTD does not change upon eLOTUS domain binding. In support of this, evidence from additional Oskar–Vasa colocalization studies indicates that the eLOTUS domain might bind to the CTD only when the helicase core is in an open state. In this assay, we tested the mutation E400Q, which resides in the DEAD box of Vasa, drastically reduces the dissociation of the ATP hydrolysis products, and consequently locks the helicase in its closed conformation bound to RNA (Xiol et al. 2014). While Oskar and wild-type Vasa co-localized in S2R+ cells (Fig. 1B), Vasa carrying the E400Q mutation did not colocalize with Oskar (Fig. 7D). The inability of Vasa E400Q to interact with Oskar in S2R+ cells is consistent with the observation that a Vasa transgene carrying the E400Q mutation is unable to localize to the posterior pole of *Drosophila* oocytes (Xiol et al. 2014), strongly suggesting that the eLOTUS domain preferentially binds the CTD when the helicase core is in an open conformation.

Taken together, the structural comparisons reveal that the eLOTUS domain binds to a specific surface on the C-terminal RecA-like domain of Vasa that is distinct from that bound by other known DEAD-box helicase stimulators.

## Discussion

Our study provides molecular insight into the function of animal LOTUS domain proteins, factors involved in diverse germline functions. We showed that the DEAD-box helicase Vasa interacts with the LOTUS domains of Oskar, TDRD5/Tejas, and TDRD7/Tapas but not with MARF1. In *Drosophila*, interaction with LOTUS domain proteins is required for Vasa localization to the nuage and germ plasm. Our structural and functional analyses of the LOTUS–Vasa interaction uncovered a key role of a C-terminal extension present in only a subset of LOTUS domains, pointing to two LOTUS domain subclasses with distinct functions in animals. The eLOTUS domain of Oskar, TDRD5, and TDRD7 not only interacts with Vasa but also stimulates its helicase activity. The mLOTUS domains present in MARF1 lack this extension and very likely have a distinct role within the germline that will need to be addressed in the future. While *Drosophila* TDRD5 (Tejas) and TDRD7 (Tapas) contain a single eLOTUS domain, some TDRD5 and TDRD7 proteins from other animals harbor mLOTUS domains in addition to their N-terminal eLOTUS domain. Whether the mLOTUS domains from MARF1, TDRD5, and TDRD7 have related activities or are functionally distinct remains to be determined.

The *Drosophila* eLOTUS domain proteins Oskar, Tejas, and Tapas have been considered to be scaffolding proteins whose function is to recruit Vasa and other germline factors to germ plasm or the nuage. While LOTUS domains were originally predicted to be RNA-binding domains (Anantharaman et al. 2010), we were unable to detect any RNA-binding activity of the eLOTUS domain of Oskar (Jeske et al. 2015). Our present study uncovered a conserved function of eLOTUS domains in binding and stimulating a DEAD-box RNA helicase, thus attributing an active regulatory role to Oskar, Tejas, and Tapas in the germline. The stimulation of the ATPase activity of Vasa by the eLOTUS domain seems universal, but its consequence and function within the germline are unknown. In *Drosophila*, Vasa stimulation by Tejas and/or Tapas in the nuage might be involved in the piRNA pathway (see below), whereas Vasa stimulation by Oskar in the pole plasm likely has a distinct role. Vasa was suggested to activate translation of mRNAs in the egg chamber through recruitment of eIF5B (Carrera et al. 2000; Johnstone and Lasko 2004), which catalyzes ribosomal subunit joining to form elongation-competent ribosomes (Pestova et al. 2000). Vasa has been shown to physically interact with eIF5B in yeast two-hybrid assays and pull-down experiments from lysates (Johnstone and Lasko 2004). A Vasa region that extends C-terminally from the helicase core was shown to be required for the eIF5B interaction (Supplemental Fig. S7A; Johnstone and Lasko 2004), which raised the question of whether eLOTUS and eIF5B jointly or mutually exclusively bind to Vasa. We aimed to test this in GST pull-down assays with recombinant proteins. However, to our surprise, we were not able to detect an interaction of Vasa with GST-eIF5B or any change in Vasa's ATPase activity in the presence of eIF5B (Supplemental Fig. S7B,C). We conclude that Vasa and eIF5B do not physically interact and that the recruitment of eIF5B by Vasa might be mediated through RNA or other proteins. It is equally plausible that Vasa's role in translation might be that of a DEAD-box RNA helicase involved in remodeling RNA–protein complexes. Given its importance in germline biology, the mechanism by which Vasa promotes translation of mRNAs merits thorough re-examination.

In the nuage, Vasa is essential for the secondary piRNA biogenesis pathway, also known as the Ping-Pong cycle (Malone et al. 2009). *Bombyx* Vasa associates with the Piwi proteins Siwi and Ago3 (Xiol et al. 2014; Nishida et al. 2015), two major players in the Ping-Pong cycle in the germ plasm (Gao and Arkov 2013; Luteijn and Ketting 2013; Sato and Siomi 2013; Czech and Hannon 2016). Within the Ping-Pong cycle, Siwi is loaded with piRNAs, and the complex binds and cleaves transposon mRNAs in an orientation antisense to piRNAs. The cleavage products are then loaded into Ago3, and the complex recognizes and cleaves piRNA cluster transcripts, leading to specific amplification of piRNAs that target transposon mRNAs present in the cell. Vasa is required for the safe handover of transposon mRNA fragments from Siwi to Ago3 (Xiol et al. 2014). Furthermore, the ATPase activity of Vasa is necessary for the release of transposon RNAs from Siwi–piRNA complexes after cleavage (Nishida et al. 2015). It is therefore possible that stimulation of Vasa by the Tejas and/or Tapas eLOTUS domains is required for high efficiency of the Ping-Pong cycle. The higher activity of Tejas compared with Tapas that we detected in our assays might be reflected in vivo by its dominant role in transposon silencing within the nuage (Patil et al. 2014).

LOTUS domains are not restricted to animals but are also present in bacteria, fungi, and plants (Anantharaman et al. 2010; Callebaut and Mornon 2010)—organisms without a Vasa ortholog. From sequence alignments, it appears that bacterial, fungal, and plant LOTUS domains lack the particular C-terminal extension, and it will be interesting to investigate and compare their function with that of mLOTUS domains of animal proteins, such as MARF1.

## Materials and methods

### Cloning and purification of recombinant proteins

*Drosophila* Oskar and Vasa sequences were amplified from genes that were codon-optimized for *Escherichia coli* using primers introducing the required restriction sites. The sequences of the LOTUS domains of Tejas and Tapas were amplified from cDNA (Tapas cDNA obtained from the *Drosophila* Genomics Resource Center [DGRC]) with primers introducing the required restriction sites. The sequences of the eLOTUS domain (the most N-terminal LOTUS domain) of *Bombyx* TDRD7 and human TDRD5 and TDRD7 were amplified from synthetic gene fragments (gBlocks purchased from IDT) codon-optimized for *E. coli* using primers introducing the required restriction sites. Sequences of the eIF5B constructs were amplified from an eIF5B plasmid obtained from Paul Lasko. All constructs were verified by sequencing. Proteins were expressed in and purified from *E. coli* (Rosetta 2) cells. The *Bombyx mori* Vasa protein sample comprised residues 135–564 and was a kind gift from Leo Nesme and Teresa Carlomagno. Details of the generation of recombinant proteins used in this study are provided in the Supplemental Material.

### Crystallization and structure determination

For crystallization, Oskar 139–240 and Vasa 463–623 were mixed in a 2:1 molar ratio to obtain a 35 mg/mL protein mixture in crystallization buffer (20 mM Tris at pH 7.5, 150 mM sodium chloride, 5 mM DTT). Equal volumes of protein complex and reservoir solution (100 mM Tris at pH 7.5, 200 mM potassium thiocyanate, 16% PEG 3350) were mixed and subjected to the hanging drop vapor diffusion method. Crystals appeared the same day at 18°C and were flash-frozen 1 wk later, and diffraction data were collected at the ID23-1 beam line of the European Synchrotron Radiation Facility (Grenoble, France). The structure was solved by molecular replacement using PHASER (McCoy et al. 2007). Search models were created from the LOTUS domain of Oskar (Jeske et al. 2015) and the helicase core of Vasa (Sengoku et al. 2006), respectively. The structure was refined using Coot (Emsley and Cowtan 2004) and PHENIX Refine (Afonine et al. 2012). Structure figures were generated using PyMol.

The crystal structure of the Oskar LOTUS–Vasa-CTD complex has been deposited in the Protein Data Bank under the accession number 5NT7.

### Protein–protein interaction assays

GST pull-down assays were performed as described (Jeske et al. 2015) with protein concentrations as indicated in the figure legends. Yeast two-hybrid experiments and plasmids coding for Vasa 1–661 (full length) and Oskar 139–606 (Short Oskar) were described previously (Jeske et al. 2015). DNA fragments coding for Vasa 1–200, Vasa 200–459 (NTD), or Vasa 460–661 (CTD) were cloned into the BamHI/EcoRI site of the pPR3-N vector. ITC measurements were performed at 25°C using a VP-ITC calorimeter (MicroCal) in buffer containing 20 mM Tris (pH 7.5) and 150 mM sodium chloride. The ITC data were corrected for the dilution heat and fitted with the Origin 7.0 software package (MicroCal).

### ATPase assays

Proteins were incubated at 23°C with 0.1 µL (corresponding to 16.6 nM) of 10 µCi/µL [γ-^32^P] ATP (Hartmann Analytic, SRP-301) in a volume of 20 µL in the presence or absence of 10 µM RNA oligo of the sequence (AGCACCGUAAAGC)_2_. The protein concentrations used are indicated in the figure legends. Per time point, 4 µL of the reaction was transferred into 50 µL of 5 mM EDTA (pH 7) to stop the reaction. The mixture was subjected to phenol/chloroform extraction, and 2 µL of the aqueous phase were analyzed by TLC using polyethyleneimine-cellulose (Merck) and 1 M formic acid and 0.5 M LiCl as solvent. The thin layer plates were analyzed by phosphorimaging, ATP hydrolysis was quantified using Fiji, and the data were fitted using SigmaPlot.

### Analysis of the transgenic *Drosophila* lines

AttB vectors containing the EGFP fusion of Vasa with or without the F504E mutation were used for ΦC31 integrase-mediated transgenesis. Transgenes were studied in the wild-type (*w*^*1118*^) or *vasa*^*PD*^/*vasa*^*D1*^ background. Details of the generation of the transgenic fly lines are provided in the Supplemental Material. Hatching assays were performed as described (Vanzo and Ephrussi 2002). For immunofluorescence, young females were kept on yeast for 2 d at 25°C, and ovaries were dissected in PBS and mounted on a coverslip. Live images were captured immediately using a 20× objective and a Zeiss LSM 780 confocal microscope and processed with Fiji. For Western blot analysis, 10 pairs of ovaries were dissected from young transgenic females that were kept on yeast for 2 d at 25°C. Ovaries were homogenized with a pestle in lysis buffer (20 mM Tris HCl at pH 7.5, 500 mM lithium chloride, 0,5% lithium dodecyl sulfate, 1 mM EDTA), and the resulting lysates were cleared in two rounds of centrifugation at 16,100*g* for 30 min at 4°C. The supernatant was mixed with SDS loading buffer, and material from approximately one-third of an ovary pair was loaded per lane of an SDS gel. Western blots were developed according to standard methods using the following antibodies: rat anti-Vasa (1:2000) (Tomancak et al. 1998), rabbit anti-GFP (1:2000; Torrey Pines Biolabs, TP401), and mouse anti-Tubulin (1:10,000; Sigma, T5168).

### Colocalization assays in cultured *Drosophila* S2R+ cells

The pAc5.1B-EGFP plasmid was a kind gift from Elisa Izaurralde. The pAc5.1B-mCherry plasmid was generated by replacing the EGFP with the mCherry sequence using the KpnI/HindIII sites. The sequence of Short Oskar (amino acids 139–606), full-length Vasa, Vasa truncations (amino acids 1–200, 200–459, and 460–661), MARF1, Tejas, Tapas, and Belle were amplified from *Drosophila* cDNA (MARF1 and Tapas cDNAs obtained from the DGRC) and cloned into the blunt-end EcoRV site of either the pAc5.1B-EGFP or pAc5.1B-mCherry vector. Vasa E400Q and Short Oskar R266E constructs were obtained by site-directed mutagenesis of the respective templates. Oskar, Tejas, and Tapas constructs lacking the LOTUS domain were generated by amplification of the vector omitting the LOTUS sequence using phosphorylated primers and religation of the PCR product. The correct sequence of all constructs was verified by sequencing.

S2R+ cells (obtained from the DGRC) were grown in Schneider's *Drosophila* medium + (L)-glutamine (Thermo Fisher Scientific) supplemented with 10% fetal bovine serum Gold (PAA) and 100 µ/mL penicillin–streptomycin (Thermo Scientific). Cells (1.5 mL) were seeded in glass-bottomed six-well plates and cotransfected with 0.2 µg of each of the respective EGFP and mCherry plasmids using the Effectene transfection reagent (Qiagen) according to the instruction manual. After 2 d, images of the live cells were taken with a 63× oil objective and a Zeiss LSM 780 confocal microscope and processed with Fiji.

## Supplementary Material

Supplemental Material
