# Supplementary material for: The LOTUS domain is a conserved DEAD-box RNA helicase regulator essential for the recruitment of Vasa to the germ plasm and nuage
Source: Genes Dev. 2017 May 1;31(9):939–52. doi: 10.1101/gad.297051.117 (PMC5458760; doi:10.1101/gad.297051.117)
Supplement: Supplemental Material [file supp_31_9_939__index.html]

The LOTUS domain is a conserved DEAD-box RNA helicase regulator essential for the recruitment of Vasa to the germ plasm and nuage — Supplemental Material 

# The LOTUS domain is a conserved DEAD-box RNA helicase regulator essential for the recruitment of Vasa to the germ plasm and nuage

## Supplemental Material

- Supplemental\_Figures.pdf
