# Supplementary material for: The LOTUS domain is a conserved DEAD-box RNA helicase regulator essential for the recruitment of Vasa to the germ plasm and nuage
Source: Genes Dev. 2017 May 1;31(9):939–52. doi: 10.1101/gad.297051.117 (PMC5458760; doi:10.1101/gad.297051.117)
Supplement: Supplemental Material [file supp_gad.297051.117_Supplemental_Figures.pdf]

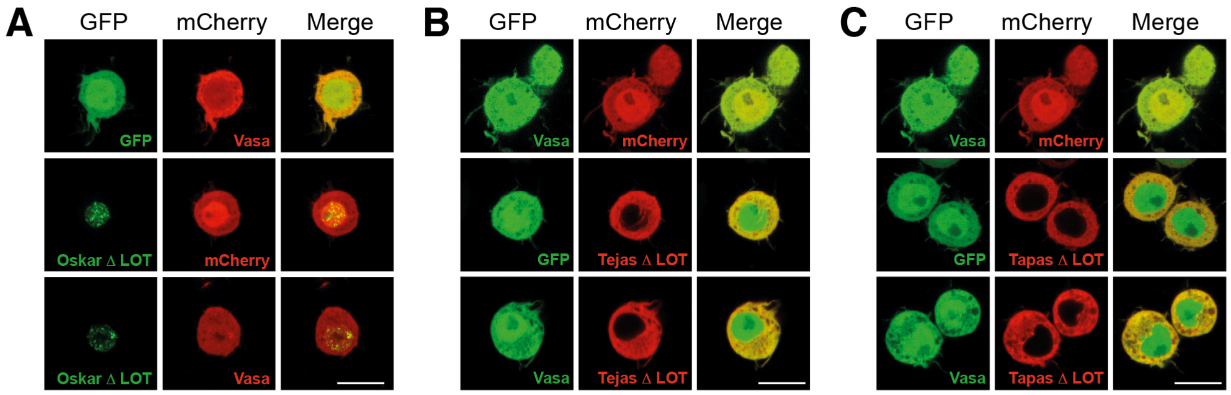

Supplemental Figure S1 (related to Figure 1). The LOTUS domain of Oskar, Tejas, and Tapas is required for co-localization with Vasa
Plasmids encoding N-terminal EGFP- or mCherry-protein fusions under the control of the actin 5C promoter were co-transfected into *Drosophila* S2R+ cells, grown for two days and imaged by confocal microscopy. Full-length proteins (in case of Oskar the short isoform) or protein constructs lacking the respective LOTUS domains (ΔLOT) were used. The scale bars indicate 10 μm.

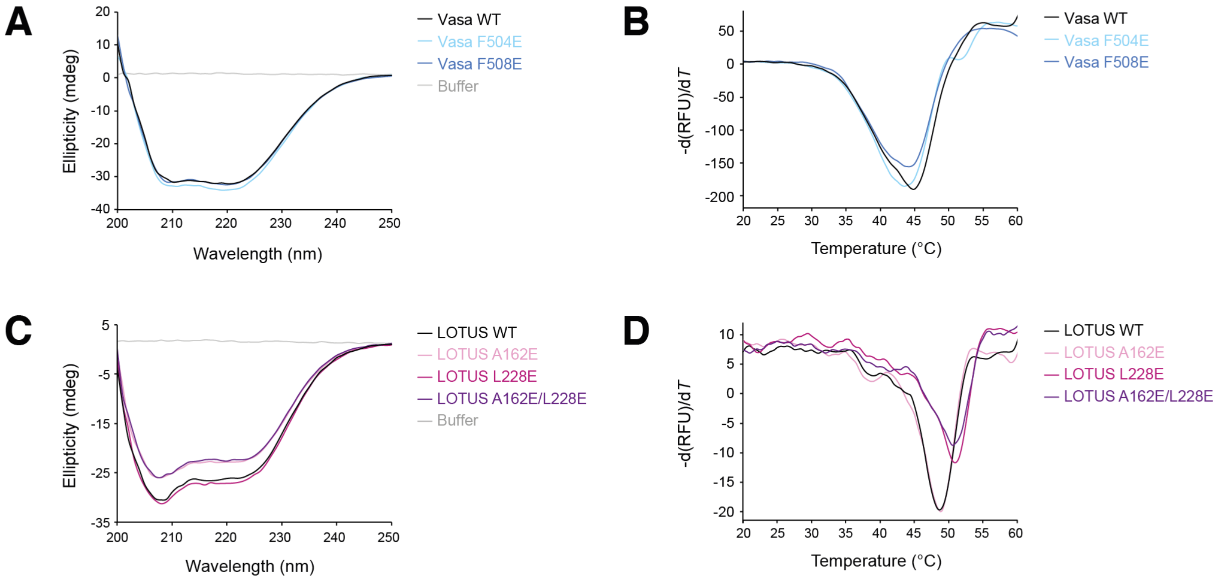

Supplemental Figure S2 (related to Figure 4). Stability of Vasa and LOTUS mutants

(A) CD spectrum of 0.2 mg/ml purified wild-type or mutant His-Vasa 200-661 (helicase core).

(B) Melt peaks derived from melt curves recorded by ThermoFluor analysis of 8 µM wild-type and mutant His-Vasa 200-661 proteins.

(C) CD spectrum of 0.2 mg/ml purified wild-type or mutant His-Oskar 139-240 (LOTUS).

(D) Melt peaks derived from melt curves recorded by ThermoFluor analysis of 8 µM wild-type and mutant His-Oskar 139-240 proteins.

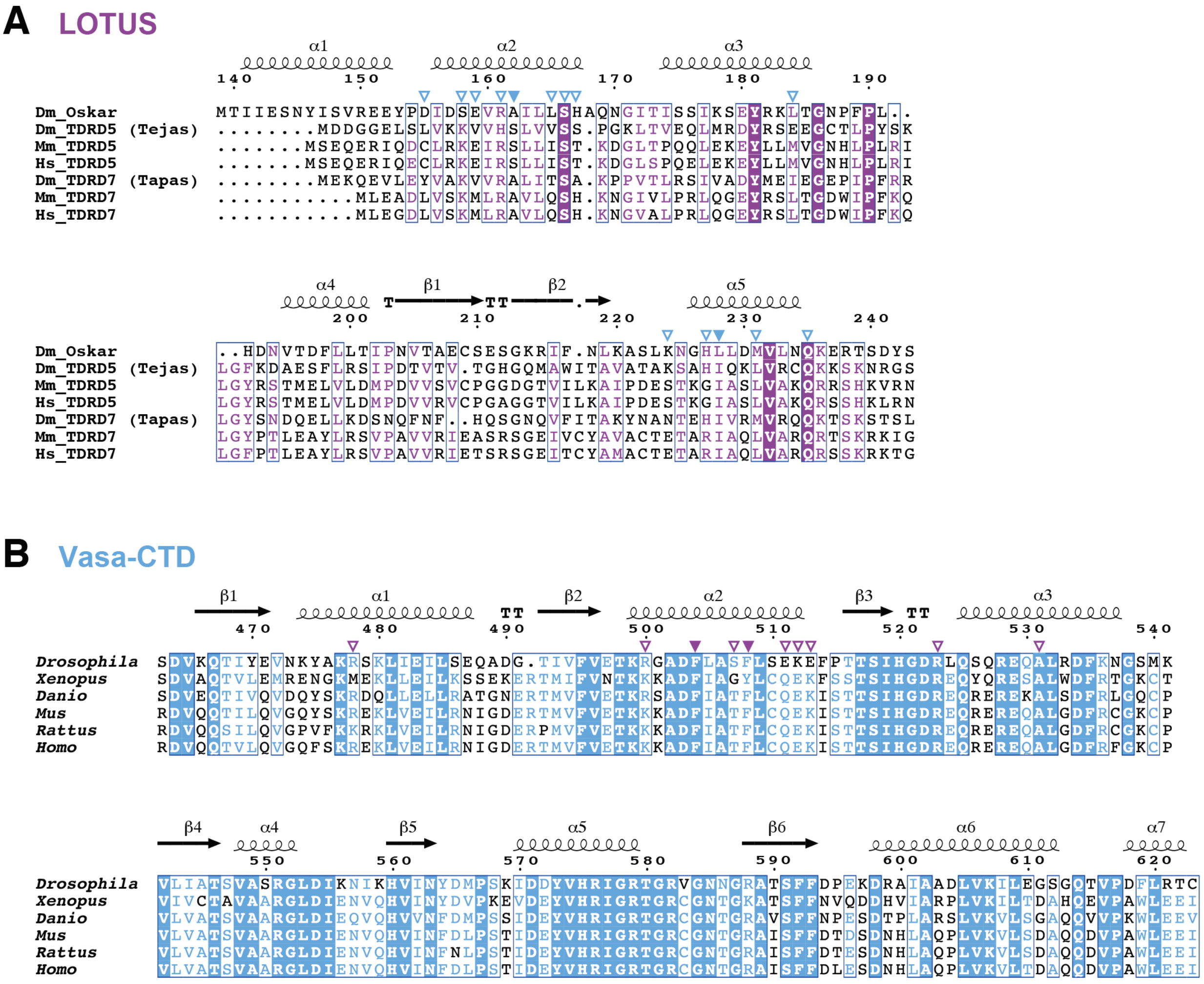

Supplemental Figure S3 (related to Figure 4). Sequence alignments of the LOTUS domain and the C-terminal RecA-like domain of Vasa

Sequence alignments were prepared using MUSCLE (Edgar 2004) and visualized with the help of ESPript (Robert and Gouet 2014; http://espript.ibcp.fr).

(A) Sequence alignment between the eLOTUS domain of *Drosophila* Oskar (residue numbering refers to the long Oskar isoform) and *Drosophila* (Dm), mouse (Mm), or human (Hs) TDRD5 and TDRD7. Secondary structure features of the eLOTUS domain of Oskar are depicted above the alignment. Residues that establish side chain-specific contacts to Vasa are highlighted as triangles above the alignment. The filled triangles indicate the residues mutated in this study.

(B) Sequence alignment of the C-terminal RecA-like domains of Vasa proteins of *Drosophila melanogaster* and indicated species. The secondary structure features depicted above the alignment refer to the *Drosophila* protein. Residues that establish side chain-specific contacts to the eLOTUS domain of Oskar are highlighted as triangles above the alignment. The filled triangles indicate the residues mutated in this study.

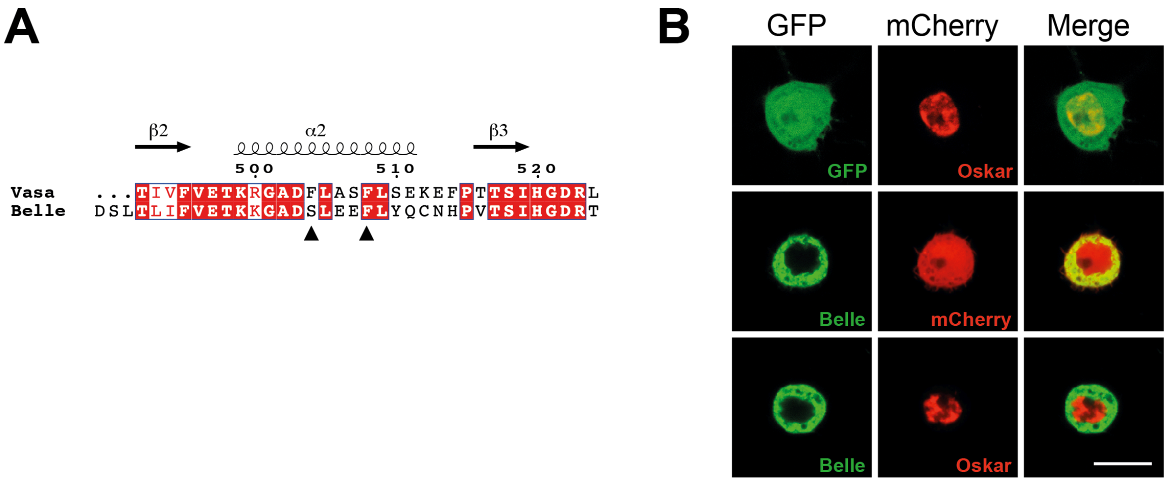

Supplemental Figure S4 (related to Figure 4). The DEAD-box helicase Belle does not interact with Oskar

(A) Sequence alignment of the eLOTUS-binding α2 helix of Vasa and the corresponding α helix of Belle. The arrowheads indicate the position of residues F594 and F508 of Vasa and their equivalents in Belle.

(B) Plasmids encoding N-terminal EGFP- or mCherry-protein fusions under the control of the actin 5C promoter were co-transfected into *Drosophila* S2R+ cells, grown for two days and imaged by confocal microscopy. Full-length proteins (in case of Oskar the short isoform) were used. The scale bars indicate 10 µm.

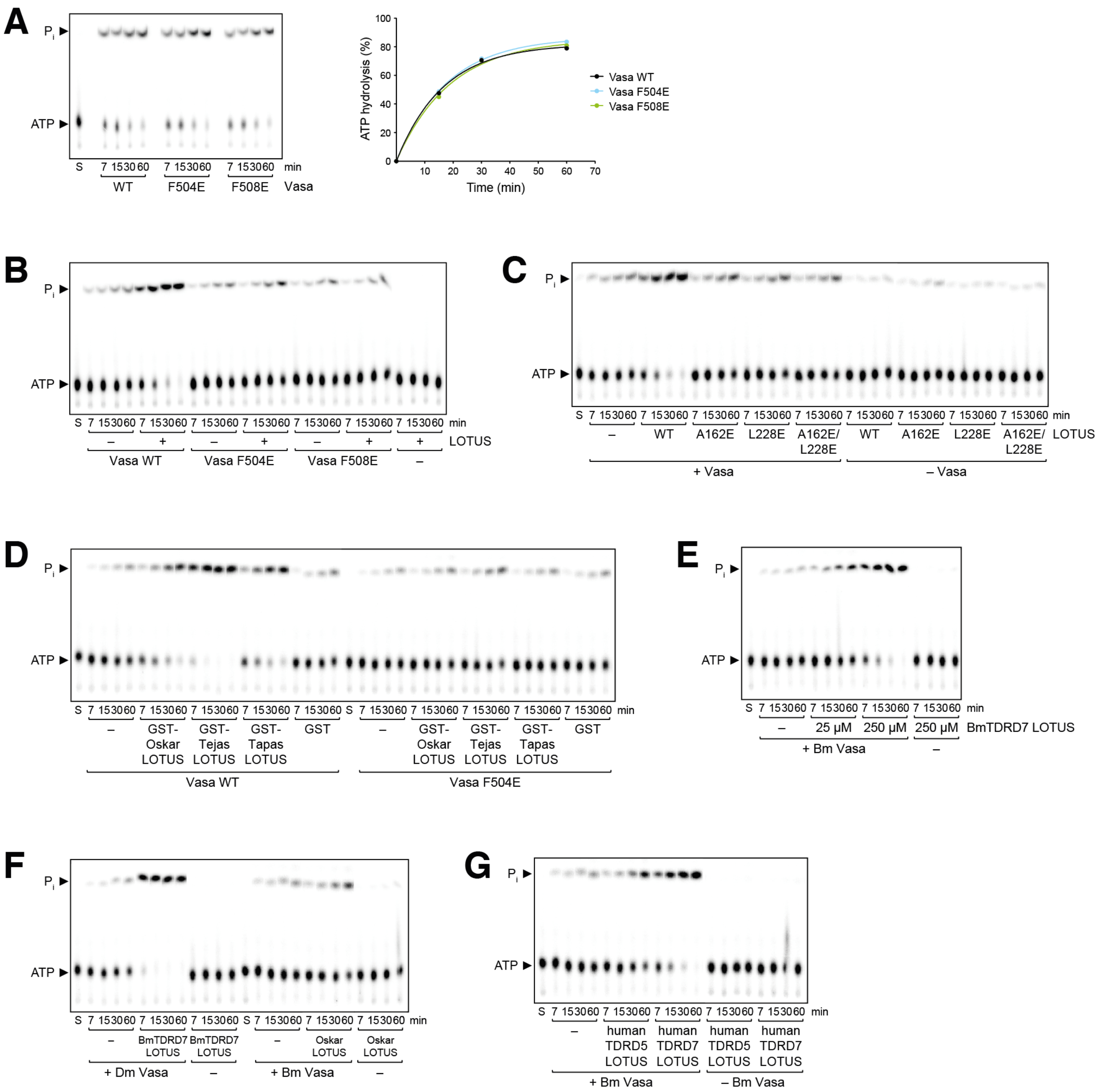

Supplemental Figure S5 (related to Figures 4 and 6). ATP hydrolysis assays

(A) ATPase time courses performed using 20 µM wild-type or mutant His-Vasa 200-661 in the presence of 30 µM RNA oligo. Original thin-layer chromatography (left panel) and quantification (right) are shown.

(B-G) Original thin-layer chromatography data from which the quantifications were derived and shown in Figures 4 and 6. Each TLC image is the result of one experiment and in some cases represents an assembly of several TLC plates that were exposed simultaneously to one phosphorimaging screen.

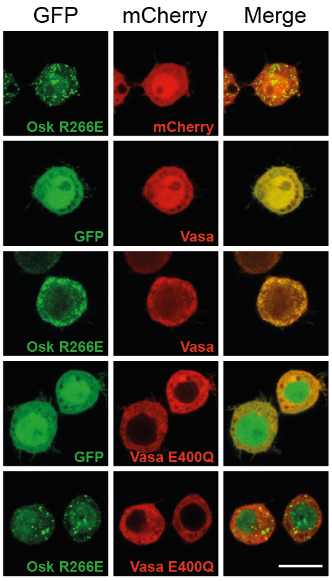

Supplemental Figure S6 (related to Figure 7). Cytoplasmic Oskar does not interact with the Vasa E400Q mutant
Plasmids encoding N-terminal EGFP- or mCherry-protein fusions under the control of the actin 5C promoter were co-transfected into *Drosophila* S2R+ cells, grown for two days and imaged by confocal microscopy. Full-length Short Oskar was mutated to contain a point mutation (R266E) in its disordered region that leads to retention of some of the protein in the cytoplasm. Full-length Vasa or E400Q mutant Vasa proteins were tested for their ability to co-localize with cytoplasmic Oskar. Whereas wild-type Vasa strongly co-localizes with this Oskar variant, mutant Vasa does not. The scale bar indicates 10 μm.

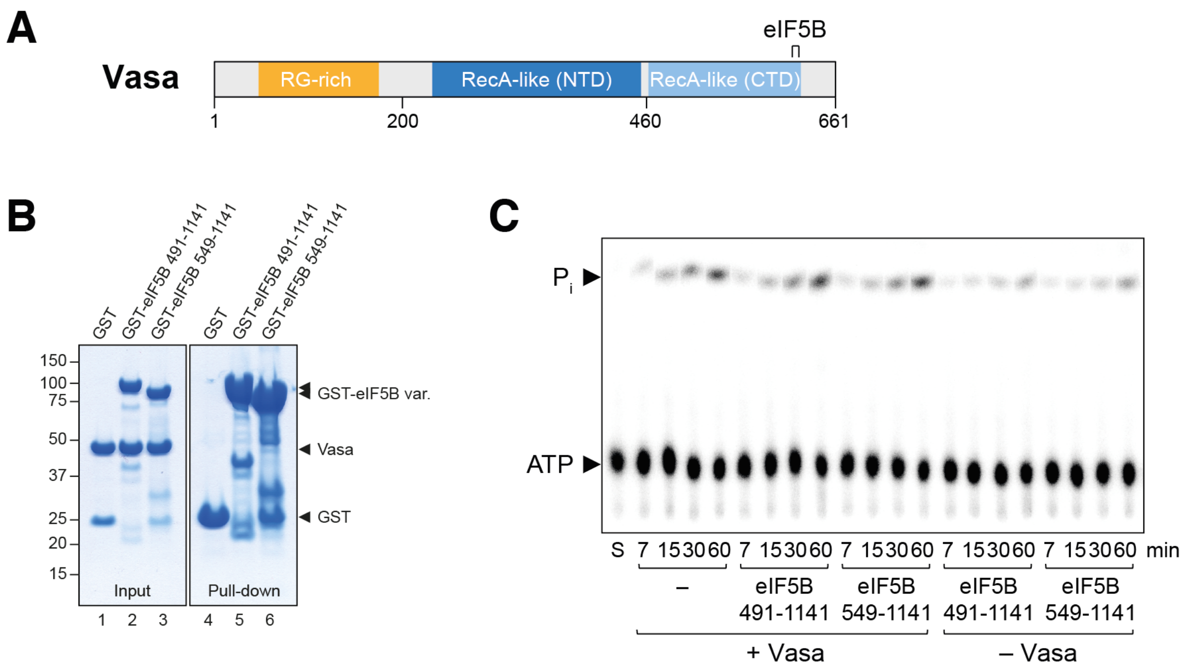

Supplemental Figure S7 (related to discussion). Vasa does not interact with eIF5B *in vitro*

(A) Domain organization of Vasa indicating the previously identified binding site for the eukaryotic translation initiation factor eIF5B (Johnstone and Lasko 2004).

(B) GST pull-down assays using 20 µM His-Vasa 200-661 and either 10 µM GST or soluble GST fusions of two different eIF5B fragments, both covering the region previously identified as interacting with Vasa in yeast-two hybrid assays (Carrera et al. 2000; Johnstone and Lasko 2004). Samples from the experiment were run on an SDS gel and stained with Coomassie Brilliant Blue. Protein markers (in kDa) are indicated on the left. No binding of Vasa to eIF5B was detected.

(C) ATPase assay using 5 µM Vasa 200-661 and 25 µM of the indicated GST-eIF5B fragments in the presence of 10 µM RNA oligo. No effect of eIF5B on the helicase activity of Vasa was detected.

Supplemental Table S1 (related to Figure 2). Data collection and refinement statistics

**Data collection**	**Osk 139-240 – Vas 463-623 complex Native**
Beamline	ID23-1 (ESRF)
Wavelength (Å)	0.97908
Space group	*P*1
Unit cell parameters	
a, b, c (Å)	38.95, 39.04, 97.48
α, β, γ (°)	89.285, 91.846, 99.262
Resolution (Å)[a]	50.0-1.4 (1.48-1.4)
R_{merge} (%)	4.8 (138.2)
I/σ(I)	10.44 (0.74)
$CC_{½}$[b]	0.999 (0.394)
Unique reflections	102457
Completeness (%)	91.4 (87.5)
Multiplicity	2.16 (2.18)
**Refinement statistics**	
Resolution (Å)	48.7-1.4
No. of reflections	102415
R_{work}/R_{free} (%)	16.9/19.9
No. of monomers in asymmetric unit	4
No. of non-H atoms	
Total	4466
Protein	3853
Water	613
Average B-factors ($Å^2$)	
Total	30.4
Protein	27.9
Water	46.2
Residues in Ramachandran plot (%)	
Favored	98.1
Allowed	1.9
Disallowed	0
R.m.s. deviations	
Bond lengths (Å)	0.007
Bond angles (°)	1.009
PDB code	5NT7

[a] Highest resolution shell is shown in parentheses.
[b] Resolution cutoff criteria according to Karplus and Diederichs 2012.

SUPPLEMENTAL EXPERIMENAL PROCEDURES

Cloning and purification of recombinant proteins

Details of generation of recombinant proteins used in this study are provided in the following table:

Construct	Vector, insertion sites	Purification steps	Application
Osk 139-240<br>(Oskar LOTUS domain)	pGEX-6P-1,<br>5’ BamHI<br>3’ NotI (blunt)	GSH sepharose,<br>His-TEV cleavage,<br>HiTrap Q<br>Gel filtration	Crystallization
Osk 144-240	His-MCN,<br>5’ NdeI (blunt)<br>3’ XbaI	Ni^{2+}-NTA agarose,<br>His-TEV cleavage,<br>HiTrap Q,<br>Gel filtration	ATPase assay
His-Osk 139-240<br>(LOTUS)	His-MCN,<br>5’ NdeI (blunt)<br>3’ XbaI	Ni^{2+}-NTA agarose,<br>Heparin,<br>Gel filtration	ATPase assay,<br>CD spectroscopy,<br>ThermoFluor
His-Osk 139-240<br>(LOTUS) A162E	Site-directed mutagenesis of His-MCN-Osk 139-240 vector		
His-Osk 139-240<br>(LOTUS) L228E			
His-Osk 139-240<br>(LOTUS) A162E/L228E			
Vasa 463-623<br>(CTD)	His-MCN,<br>5’ NdeI (blunt)<br>3’ XbaI	Ni^{2+}-NTA agarose,<br>His-TEV cleavage,<br>HiTrap SP,<br>Gel filtration	Crystallization
Vasa 200-661		Ni^{2+}-NTA agarose,<br>His-TEV cleavage,<br>Heparin,<br>Gel filtration	GST pull-down assay,<br>ATPase assay
His-Vasa 200-661<br>(Vasa WT)		Ni^{2+}-NTA agarose<br>Heparin<br>Gel filtration	GST pull-down assay,<br>ATPase assay,<br>CD spectroscopy,<br>ThermoFluor
His-Vasa 200-661<br>F504E<br>(Vasa F504E)	Site-directed mutagenesis of His-MCN-Vasa 200-661 vector		
His-Vasa 200-661<br>F508E<br>(Vasa F508E)			
His-BmTDRD7 1-100<br>(BmTDRD7 LOTUS)	His-MCN,<br>5’ NdeI (blunt)<br>3’ XbaI	Ni^{2+}-NTA agarose,<br>Heparin,<br>Gel filtration	ATPase assay
His-HsTDRD5 1-97<br>(HsTDRD5 LOTUS)		Ni^{2+}-NTA agarose,<br>HiTrap Q,<br>Gel filtration	

His-Hs TDRD7 1-93<br>(HsTDRD7 LOTUS)		Ni^{2+}-NTA agarose,<br>Heparin,<br>Gel filtration	
GST	Expressed from pGEX-6P-1	GSH sepharose,<br>HiTrap Q,<br>Gel filtration	GST pull-down assay,<br>ATPase assay
GST-Osk 139-240<br>(GST-Oskar LOTUS)	pGEX-6P-1,<br>5' BamHI<br>3' NotI (blunt)		
GST-Osk 139-222<br>(GST-LOTUS ∆C)		GSH sepharose,<br>Gel filtration	GST pull-down assay
GST-Osk 139-240 A162E<br>(GST-LOTUS A162E)	Site-directed mutagenesis of pGEX-6P-1-Osk 139-240 vector		
GST-Osk 139-240 L228E<br>(GST-LOTUS L228E)			
GST-Osk 139-240 A162E/L228E<br>(GST-LOTUS A162E/L228E)			
GST-Tejas 1-100<br>(GST-Tejas LOTUS)	pGEX-6P-1,<br>5' BamHI<br>3' NotI (blunt)	GSH sepharose,<br>Heparin,<br>Gel filtration	GST pull-down assay,<br>ATPase assay
GST-Tapas 1-100<br>(GST-Tapas LOTUS)			
GST-eIF5B 491-1141		GSH sepharose,<br>HiTrap Q,<br>Gel filtration	
GST-eIF5B 549-1141			

Biophysical measurements

Circular dichroism spectra of the proteins (in 20 mM Tris pH 7.5, 150 mM sodium chloride, 10% glycerol) were recorded on a Jasco J-715 spectropolarimeter at 20°C in a Hellma quartz cuvette (0.2 cm path length). The ThermoFluor assays (Boivin et al. 2013) were performed with a StepOne Plus Real-Time PCR system (Applied Biosystems). Proteins (in 20 mM Tris pH 7.5, 150 mM sodium chloride, 10% glycerol) were mixed with SYPRO Orange (Invitrogen, S6651) in a total volume of 25 µl. The samples were subjected to a temperature gradient from 20°C to 98°C over the period of 2 hours and the emitted fluorescence signal (λ=590 nm) was recorded simultaneously.

Generation of transgenic *Drosophila* lines

The *K10* signal sequence was removed from the pUAS-K10attB vector (Koch et al. 2009) by *Nde*I/*Xba*I digestion, filling in the 5' overhangs and re-ligating the blunt ends. The modified attB vector was digested with *Bam*HI, 5' overhangs were filled and the product was digested with *Xho*I, which resulted in removal of the UAS promoter sequence. A DNA fragment covering the *vasa* promoter, a multiple cloning site, and the *vasa* 3' UTR was amplified from the pVasa-MCS-vasa-(3x3P-EGFP)-attB vector (kind gift of Ramesh Pillai) with primers introducing a 5' *Xho*I and a 3' *Sma*I site and ligated into the *Xho*I/blunt sites of the modified attB vector resulting in the Pvasa-MCS-vas3'UTR-attB vector. mEGFP was amplified using primers introducing a 5' *Kpn*I and a

3’ *Avr*II site and the PCR product was inserted into the corresponding sites in the Pvasa-MCS-vas3’UTR-attB vector, resulting in the Pvasa-EGFP-MCS-vas3’UTR-attB vector. The coding sequence of *vasa* was subcloned into the PCR Blunt II-TOPO vector (Life technologies) and subjected to site-directed mutagenesis to introduce the F504E mutation. Wild-type or mutated *vasa* sequences were amplified from the TOPO vector introducing a 5’ *Avr*II and a 3’ *Asc*I site and inserted into the corresponding sites in the MCS of the Pvasa-EGFP-MCS-vas3’UTR-attB vector. The resulting *vasa*-attB vectors were integrated into position 65B2 (3L) of the genome of the VK33 fly strain using ΦC31 integrase. Transgenes were studied in a wild-type (w^{1118}) or $vasa^{PD}/vasa^{D1}$ background.

SUPPLEMENTAL REFERENCES

Boivin S, Kozak S, Meijers R. 2013. Optimization of protein purification and characterization using Thermofluor screens. *Protein Expr Purif* **91**: 192–206.

Edgar RC. 2004. MUSCLE: Multiple sequence alignment with high accuracy and high throughput. *Nucleic Acids Res* **32**: 1792–1797.

Karplus P A., Diederichs K. 2012. Linking Crystallographic Model and Data Quality. *Science* **336**: 1030–1033.

Koch R, Ledermann R, Urwyler O, Heller M, Suter B. 2009. Systematic functional analysis of Bicaudal-D serine phosphorylation and intragenic suppression of a female sterile allele of BicD. *PLoS One* **4**.

Robert X, Gouet P. 2014. Deciphering key features in protein structures with the new ENDscript server. *Nucleic Acids Res* **42**: W320–W324.
